# Network-Informed Constrained Divisive Pooled Testing Assignments

**DOI:** 10.3389/fdata.2022.893760

**Published:** 2022-07-08

**Authors:** Daniel K. Sewell

**Affiliations:** Department of Biostatistics, University of Iowa, Iowa City, IA, United States

**Keywords:** group testing, infectious disease, network analysis, divisive clustering, epidemiology

## Abstract

Frequent universal testing in a finite population is an effective approach to preventing large infectious disease outbreaks. Yet when the target group has many constituents, this strategy can be cost prohibitive. One approach to alleviate the resource burden is to group multiple individual tests into one unit in order to determine if further tests at the individual level are necessary. This approach, referred to as a group testing or pooled testing, has received much attention in finding the minimum cost pooling strategy. Existing approaches, however, assume either independence or very simple dependence structures between individuals. This assumption ignores the fact that in the context of infectious diseases there is an underlying transmission network that connects individuals. We develop a constrained divisive hierarchical clustering algorithm that assigns individuals to pools based on the contact patterns between individuals. In a simulation study based on real networks, we show the benefits of using our proposed approach compared to random assignments even when the network is imperfectly measured and there is a high degree of missingness in the data.

## 1. Introduction

The silent spreading of an infectious disease occurs when individuals who are asymptomatic or presymptomatic transmit the disease to those who are not infected. This has been one of the defining features of the current COVID-19 pandemic, differentiating SARS-CoV-2 from, say, the 2003 SARS-CoV epidemic (Huff, 2020). Many studies have shown COVID-19 asymptomatic rates of 50% or higher (Oran and Topol, 2020; Sutton et al., 2020; Almadhi et al., 2021), and even when symptoms do appear, peak viral shedding occurs prior to the presentation of symptoms (He et al., 2020). Researchers have noted that even isolating 100% of symptomatic cases at the time of symptom onset is insufficient for infection control (Moghadas et al., 2020), noting that “current strategies that rely solely on ‘symptom onset' for infection identification need urgent reassessment” (Huff and Singh, 2020).

There are two traditional methods of dampening the impact of silent spread. The first is contact tracing, whereby known cases are asked to enumerate their recent contacts, and these contacts are subsequently asked to adhere to quarantining procedures. However, there exist many opportunities for this strategy to fail. Sociological studies have long shown that individuals (the known case, in our context) may forget several contacts, even some of the most important ones (Killworth and Bernard, 1976, 1977, 1979; Bernard et al., 1979, 1982; Freeman et al., 1987). In addition, it may be hard to make contact with these individuals, and even should contact be made, these individuals may choose to ignore some or all quarantining protocols. Indeed, studies have shown that the success rate of quarantining contacts in known cases is less than 20% (Reynolds et al., 2008; Bharti et al., 2020).

The second strategy for controlling silent spread is to implement regular universal screening, whereby everyone within some finite population of interest is tested on a regular basis in order to detect cases prior to symptom onset. This can be a highly efficacious strategy, but the frequency of testing often must be high (Larremore et al., 2021). This places a very large resource burden on those tasked with providing so many tests, as still seen in the COVID-19 pandemic (Huff, 2020).

Pooled testing is a method that in certain circumstances can be used to greatly alleviate this resource burden (Abdalhamid et al., 2020; Pilcher et al., 2020; Wacharapluesadee et al., 2020). In the COVID-19 pandemic, several countries have implemented pooled testing, such as China, Germany, Israel, and Thailand (Mandavilli, 2020). Within the United States, several organizations have also implemented pooled testing, including the Nebraska Public Health Laboratory (Stone, 2020), Duke University (Denny et al., 2020), Stony Brook University (The State University of New York at Stony Brook, 2020), and UC San Diego Health (Elkalla, 2020).

Broadly speaking, pooled testing is the act of combining multiple individual tests in order to determine whether individual-level testing is necessary. The analysis of pooled tests was first formalized in work by Dorfman (1943), which has since been referred to as the two-stage Dorfman procedure. This is a simple approach where a certain number of samples are pooled and tested; should the resulting diagnostic test be negative, no more tests are conducted, whereas if positive, all individuals comprising the pool are subsequently tested. Other pooled testing strategies include the Sterrett Procedure (Sterrett, 1957) as well as hierarchical approaches (Black et al., 2015; Malinovsky et al., 2020). Work has also been done to generalize these procedures to the context where there are known heterogeneous probabilities of being infected (e.g., Hwang, 1975), including some of the previously mentioned studies. Because of the simplicity and widespread use of the two-stage Dorfman procedure (Hughes-Oliver, 2006), we will focus on this pooled testing strategy.

The above approaches all depend on the assumption of independent samples. This may be reasonable in some contexts, but when in the context of infectious disease, this assumption can only be justified if those being tested are sufficiently isolated from one another. If, e.g., a school, workplace, or public health department is testing a set of individuals who interact with one another, this assumption is grossly violated. This independence assumption is relaxed in a study by Lendle et al. (2012), yet even here it is assumed that the individuals being tested are exchangeable within certain clusters, and that individuals in different clusters are independent. This may be applicable in some settings (such as the example in Lendle et al. (2012)'s study where multiple T-cell responses are measured within each individual, and hence a compound symmetry correlation structure is reasonable), but is clearly not the case with any realistic transmission network. In a recent study, Sewell (In Press) developed a method for utilizing network information in order to improve pooled testing efficiency. However, the proposed simulated annealing algorithm is very computationally burdensome and is simply not feasible for medium to large networks. The goal of this study is to develop an algorithm that can improve the efficiency of the two-stage Dorfman procedure by leveraging information on the underlying transmission network.

The remainder of the paper is as follows. Sections 2.1, 2.2 describes the objective function and our proposed algorithm. Section 2.3 describes the data we analyzed and the simulation study conducted. Section 3 reports the results from this study, and Section 4 provides a discussion.

## 2. Methods

### 2.1. Objective

It has long been recognized that in the presence of diagnostic testing error (i.e., the sensitivity and specificity do not both equal 1), it should not be the goal to only minimize the expected number of tests. Rather, the expected number of correct classifications ought to be accounted for as well. Malinovsky et al. (2016) proposed using the ratio of the expected number of correctly classified individuals to the expected number of tests and then derived this quantity for the case of independent individuals. For the more general setting, our objective function is given below, but first, we need to introduce some notation.

Let *y*_*i*_ equal one if the *i*^*th*^ individual is infected and zero otherwise for *i* = 1, 2, …, *N*, where *N* is the number of individuals to participate in the pooled testing. Let *Z*_*i*_ ∈ {1, 2, …, *P*} denote which of the *P* pools individual *i* belongs to, and let $I_{p}\subset \left\{1,\ldots ,N\right\}$ be the set of individuals belonging to the *p*^*th*^ pool, each of which is of size *K* (= *N*/*P*). Let *T* denote the total number of tests conducted and *C* the total number of correct classifications. Finally, let *p* denote the population prevalence of the disease, and let *S*_*p*_ and *S*_*e*_ denote the specificity and sensitivity of the test, respectively.

With regards to the network, let *A* denote the *N* × *N* adjacency matrix such that *A*_*ij*_ equals one if there is an edge between actors *i* and *j* and zero otherwise. Let $N_{i}$ denote the neighbors of *i*, i.e., {*j*:*A*_*ij*_ = 1}.

The expected number of tests for the *N* individuals for a given pooling assignment vector *Z* can be shown to equal


(1) $$𝔼(T|Z)=P+nS_{e}-K(S_{p}+S_{e}-1)\sum_{p=1}^{p}\mathbb{P}(y^{\prime }{}_{Ip}{𝟙}_{K}=0),$$

where 𝟙_*m*_ is the *m* × 1 vector of ones. The expected number of correct classifications given *Z* can be shown to equal


(2) $$\begin{array}{l} \begin{array}{cc} 𝔼(C|Z) & =\;nS_{e}^{2}+N(1-p)(S_{e}S_{p}+1-S_{e}-S_{e}^{2} \end{array})\\ \;+K(1-S_{p})(S_{p}+S_{e}-1)\sum_{p=1}^{p}\mathbb{P}({y^{\prime }}_{Ip}{𝟙}_{K_{p}}=0). \end{array}$$

The objective function is then defined to be


(3) $$\begin{array}{r} Q\left(Z\right):=\frac{𝔼\left(C|Z\right)}{𝔼\left(T|Z\right)} \end{array}$$

In very few cases will the quantities $\mathbb{P}(y^{\prime }{}_{Ip}{𝟙}_{K_{p}}=0)$, and hence *Q*(*Z*), be known in a closed form. However, given any arbitrary simulator *F* of a data set **y** (e.g., that of a network-based compartmental or agent-based model), we can use Monte Carlo approximations to obtain arbitrarily exact estimates of these probabilities.

### 2.2. Constrained Divisive Pool Assignments

The way in which the specific assignation of individuals to pools affects the objective function is through the probability of having pools with no infected individuals. That is, the numerator of *Q*(*Z*) is maximized and the denominator is minimized by maximizing $\sum_{p=1}^{p}\mathbb{P}(y^{\prime }{}_{Ip}{𝟙}_{K_{p}}=0)$. Telescoping this quantity out in the following way is, while very simple, somewhat revelatory to our purposes:


(4) $$\begin{array}{l} \sum_{p=1}^{P}\mathbb{P}(y^{\prime }{}_{Ip}{\text{𝟙}}_{K_{p}}=0)\\ =\sum_{p=1}^{P}\left[\mathbb{P}(y_{i_{p1}}=0)\prod_{k=2}^{K}\mathbb{P}(y_{i_{pk}}=0|y_{i_{p1}}=\cdots =y_{i_{p(k-1)}}=0)\right], \end{array}$$

where the subsequence ${\left\{i_{pk}\right\}}_{k=1}^{K}$ consists of the *K* members of $I_{p}$.

In the context of infectious disease, we feel it is eminently reasonable to assume the following:


(5) $$\begin{array}{l} \text{For}\;S_{1},S_{2}\subset \{1,\ldots ,N\}\setminus \{i\}\text{ such that}|S_{1}|\;=\;|\;S_{2}|,\\ \text{if}|S_{1}\cap N_{i}|\;>\;|S_{2}\cap N_{i}|\\ \text{then }\mathbb{P}(y_{i}=0|\{y_{j}=0,j\in S_{1}\})>\mathbb{P}(y_{i}=0|\{y_{j}=0,j\in S_{2}\}). \end{array}$$

In other words, we are more confident that an individual is not infected if we know their neighbors are also not infected than if we know that the same number of non-neighbors are not infected. As an example of this, consider the following autologistic actor attribute model (ALAAM) (Robins et al., 2001), given by:


$$\mathbb{P}\left(y\right)=\frac{1}{\phi \left(\theta \right)}\exp\left\{\theta_{1}y^{\prime }{𝟙}_{N}+\frac{\theta_{2}}{2}y^{\prime }Ay\right\},$$

which controls the overall prevalence of the disease through the parameter θ_1_ and the transmissibility between neighbors through θ_2_, and where ϕ(**θ**) is a normalizing constant involving **θ**: = (θ_1_, θ_2_). Without loss of generality, consider $\mathbb{P}(y_{1}=0|\{y_{j}=0,j\in S\})$ for some set S:={2,3,…,S}. This quantity can be shown to equal


$$\mathbb{P}(y_{1}=0|\{y_{j}=0,j\in S\})={\left[1+\frac{\sum_{\left\{y_{j},j>S\right\}}\exp\left\{\theta_{1}\sum_{j>S}y_{j}+\theta_{2}\left(\sum_{j>S}y_{j}A_{1j}+\sum_{S<j<k}y_{j}y_{k}A_{jk}\right)\right\}}{\sum_{\left\{y_{j},j>S\right\}}\exp\left\{\theta_{1}\sum_{j>S}y_{j}+\theta_{2}\sum_{S<j<k}y_{j}y_{k}A_{jk}\right\}}e^{\theta_{1}}\right]}^{-1}.$$

From this, it can be seen that the higher the proportion of actor 1's edges belong to set S, and hence the smaller the quantity $\underset{j>S}{\sum }y_{j}A_{1j}$, the larger the conditional probability that *y*_1_ = 0.

Under the mild assumption in Equation (5), it can be seen through Equation (4) that *Q*(*Z*) is maximized when the edges connect individuals in the same pool. That is, we wish to minimize the boundary sets of edges bridging individuals in different pools. To this end, we begin with spectral clustering, a natural candidate for this type of problem (refer to, e.g., Von Luxburg, 2007). However, we cannot simply apply *k*-means or some other simple clustering algorithm to the eigenvalues of the Laplacian matrix because our pool sizes are each fixed a priori at *K*. Therefore, we propose using a constrained divisive clustering method based on DIANA (MacNaughton-Smith et al., 1964; Kaufman and Rousseeuw, 1990).

Our proposed approach begins by computing the Laplacian matrix, *L*: = *D*−*A*, where *D* is the diagonal matrix with the actors' degrees along with the diagonal elements (i.e., $D_{ii}:=\underset{j}{\sum }A_{ij}$) and finding the eigenvectors corresponding to the *P* smallest eigenvalues. We then compute the distances between all *N* individuals and assign to the first pool the individual *i*_11_ who has the largest mean distance to all others. For *k* = 2, …, *K*, we find the individual *i*_1*k*_ who has the largest difference between the mean distance to those not belonging to the pool and the mean distance to those *k*−1 individuals currently assigned to the pool. We remove these individuals (*i*_11_, …, *i*_1*K*_), and then iterate this for pools 2 through *P*−1, where this last iteration splits the final 2*K* individuals into the last two pools. Details of the algorithm are given below in Algorithm 1.

**Algorithm 1:**
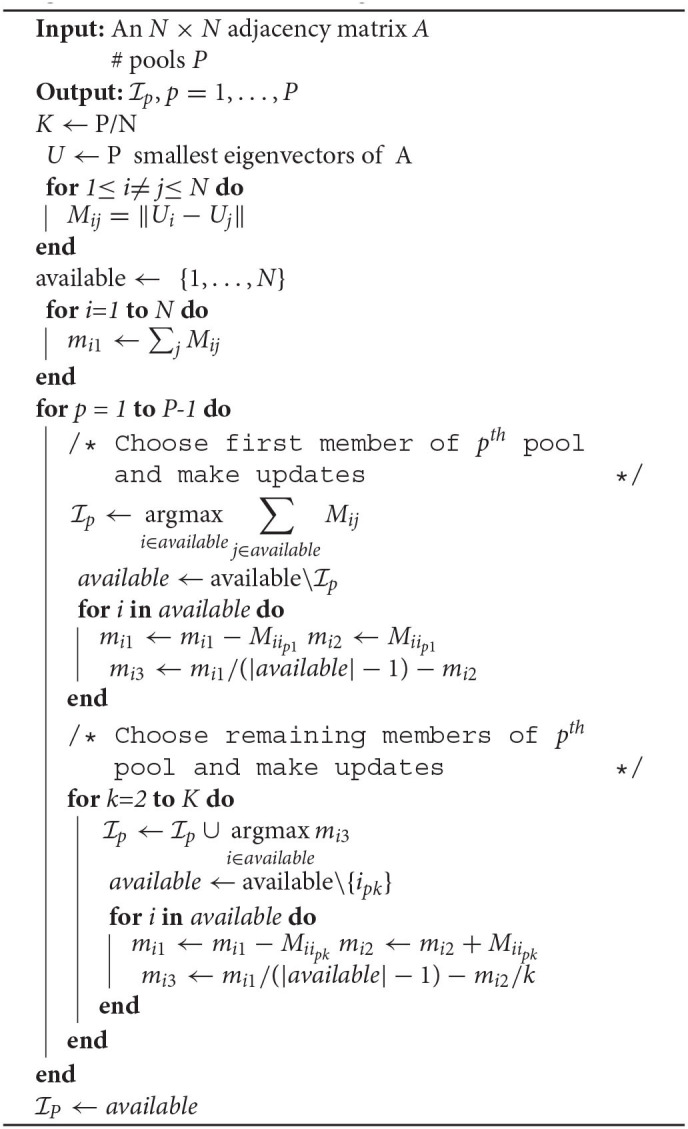
Divisive Pool Assignment Procedure.

In nearly all cases, however, the pool size *K* will be relatively small (e.g., *K*∈[1, 100]), and certainly will not grow with *N*, i.e., P=O(N). This induces a computational cost $O\left(N^{3}\right)$ that is too high for large networks. In such cases, we, therefore, suggest replacing the distances obtained from the *P* eigenvalues in Algorithm 1 with the geodesic distances, which only costs $O\left(N^{2}\right)$ to compute (Newman, 2010). We will refer to this modification as Algorithm 2.

### 2.3. Add Health Data Analysis

#### 2.3.1. Network Data

The National Longitudinal Survey of Adolescent Health (Add Health) collected information from a nationally representative sample of adolescents in grades 7 through 12 spanning 144 schools (Moody, 1999). Out of this study came friendship networks among students, which we will take to serve as a proxy for which students are most likely to transmit to one another. Data for 84 schools are available through the R package networkdata (Almquist, 2014), with networks ranging in size from 25 to 2,587 students. For our analyses, we focused on two networks, one having 495 actors and 2,675 edges, and the other having 2,587 actors and 12,969 edges.

Network survey data has often been used in infectious disease modeling (Hoang et al., 2019). Similar to contact diaries which have shown reasonably good associations between long contacts measured by sensor devices (e.g., Smieszek et al., 2014; Leecaster et al., 2016), in a study looking at high school data in France all long duration contacts were represented in a friendship network survey, and “the overall structure of the contact network […] is correctly captured by […] [self-reported] friendships” (Mastrandrea et al., 2015). While self-reported friendship data may not be sufficiently accurate in all contexts, in the context of school students there is at least reasonable evidence showing that the long contacts which are most likely to act to transmit close-contact diseases are well approximated by self-reported friendships.

To evaluate our method on larger networks, we created a synthetic network having realistic topology in the following way. We fit an exponential random graph model (ERGM) based on the social-circuit dependence assumption on each of the 84 school networks described above. More specifically, each ERGM was fit using the following terms: # edges, # 2-stars, # triangles, geometrically weighted edgewise shared partners, and geometrically weighted dyadwise shared partners. The first three terms correspond to Markov dependencies, and the latter two to the social-circuit dependencies (Lusher et al., 2012). We then performed a fixed effects meta-analysis, where each coefficient was modeled as a function of the log of the network size. Using these coefficients, we then generated a network of size 10,000 actors, having 13,800 edges. Along with the two networks of size 495 and 2,675, this then gave us a third network to analyze, and we will refer to these networks as AH495, AH2587, and ERGM10000, respectively.

#### 2.3.2. Simulation Framework

To evaluate *Q*(*Z*), we used a network-based susceptible-infectious-susceptible (SIS) model as our simulator *F* (refer to, e.g., Allen et al., 2008). In most realistic infectious disease contexts where pooled testing may be implemented, there is more knowledge of the prevalence of the disease than other facets of disease spread. Therefore, we constrained the SIS model such that the prevalence is within a small range; in the simulation results given below, we chose 0.025±0.0075. Thus, in order to get samples from *F* with which to estimate *Q*(*Z*) we repeatedly performed the following steps until the desired number of simulated datasets were obtained:

1. Draw the SIS transmission parameter from a uniform distribution.
2. Draw new *y*_*i*_, *i* = 1, …, *N* from SIS model.
3. If $\frac{1}{N}\underset{i}{\sum }y_{i}\in \left[0.025-0.0075,0.025+0.0075\right]$ accept **y**, else reject.

With *Q*(*Z*) estimated *via* Monte Carlo from these draws from *F*, we can choose the optimal pool size *K*.

We then expanded our study to determine the effect of having imperfect knowledge of the underlying network, as well as the effect of varying non-response rates. We replicated two common network survey tools in simulating data. First, we simulated open ended responses with imperfect recall rates. This *partial recall* strategy assumed each individual would “forget” a given edge with a probability of 0.25. Second, we simulated a *nominate-n* design, where each individual gets to nominate up to *n* of their edges. In our simulations, we set *n* = 5. To address non-response, we simulated “observed” networks *via* the partial recall and nominate-5 strategies with 5, 10, or 20% of the network members failing to provide responses. For each configuration, we simulated 250 networks and estimated *Q*(*Z*) for each.

## 3. Results

**Figure 1:**
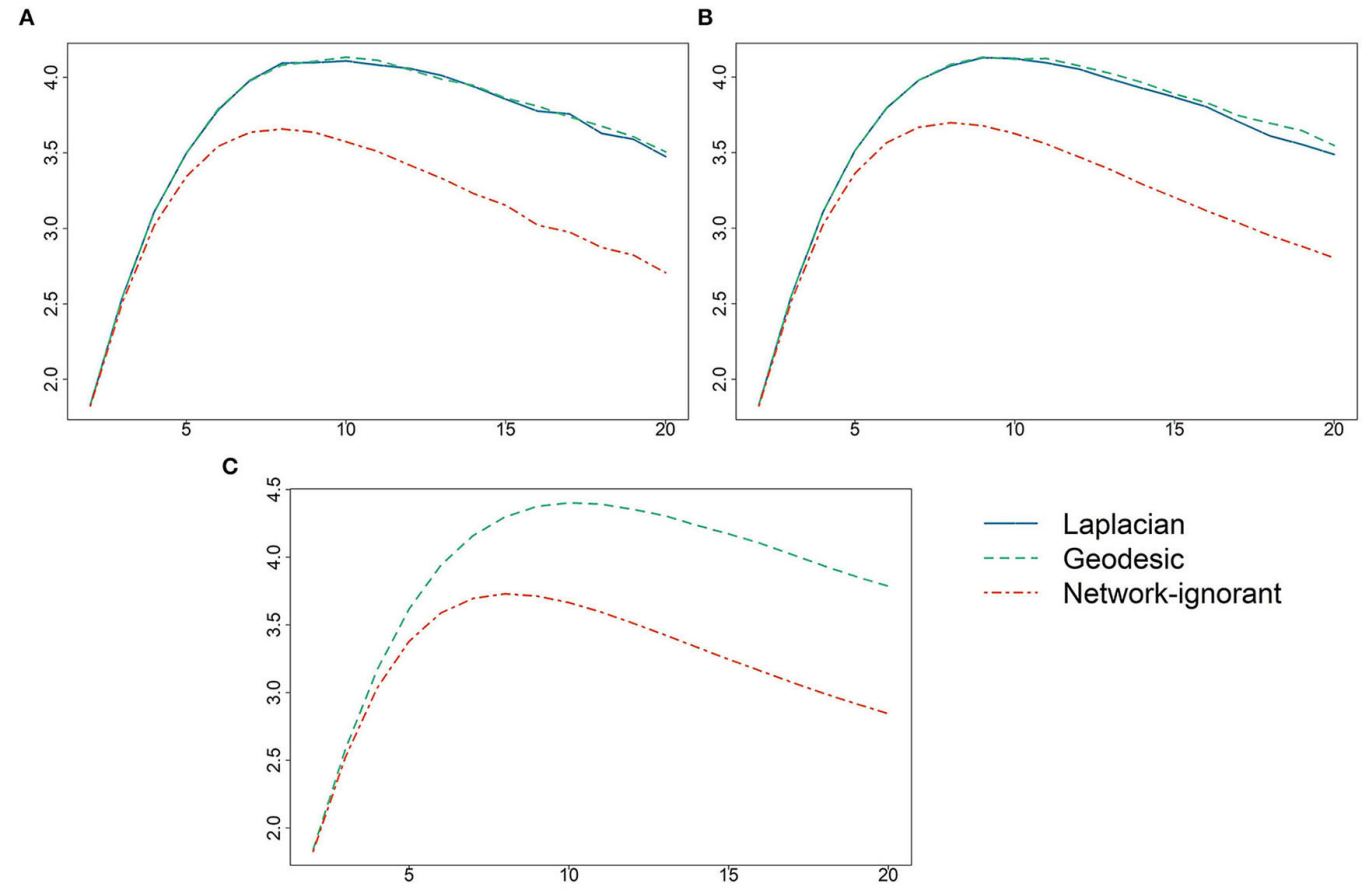
Values of the objective function *Q*(*Z*) (vertical axis) vs. the pool size *K* (horizontal axis) for **(A)** AH495, **(B)** AH2587, and **(C)** ERGM10000. Values of *Q* are given using Algorithm 1 based on the Laplacian eigenvectors, Algorithm 2 based on geodesic distances, and using random pool assignments.

The values of *Q*(*Z*) for *K* ranging from 2 to 20 are displayed in Figure 1. The optimal pool sizes for AH495, AH2587, and ERGM10000 were 10, 9, and 10, respectively. The dashed-dotted red line represents the average value of *Q* over 50 randomly assigned pools for each *K*. Results from Algorithm 1 based on the Laplacian are given in the solid blue line, and from Algorithm 2 based on geodesic distances in dashed green; for ERGM10000 it was not feasible to use Algorithm 1. It is clear that there is a negligible difference in performance between the Algorithm 1 and the more computationally efficient Algorithm 2 algorithms. Utilizing the network to inform the specific pool assignments dominated random pool assignments for all pool sizes *K*, and for all but very small pool sizes greatly increased the expected number of correct classifications per test.

**Figure 2:**
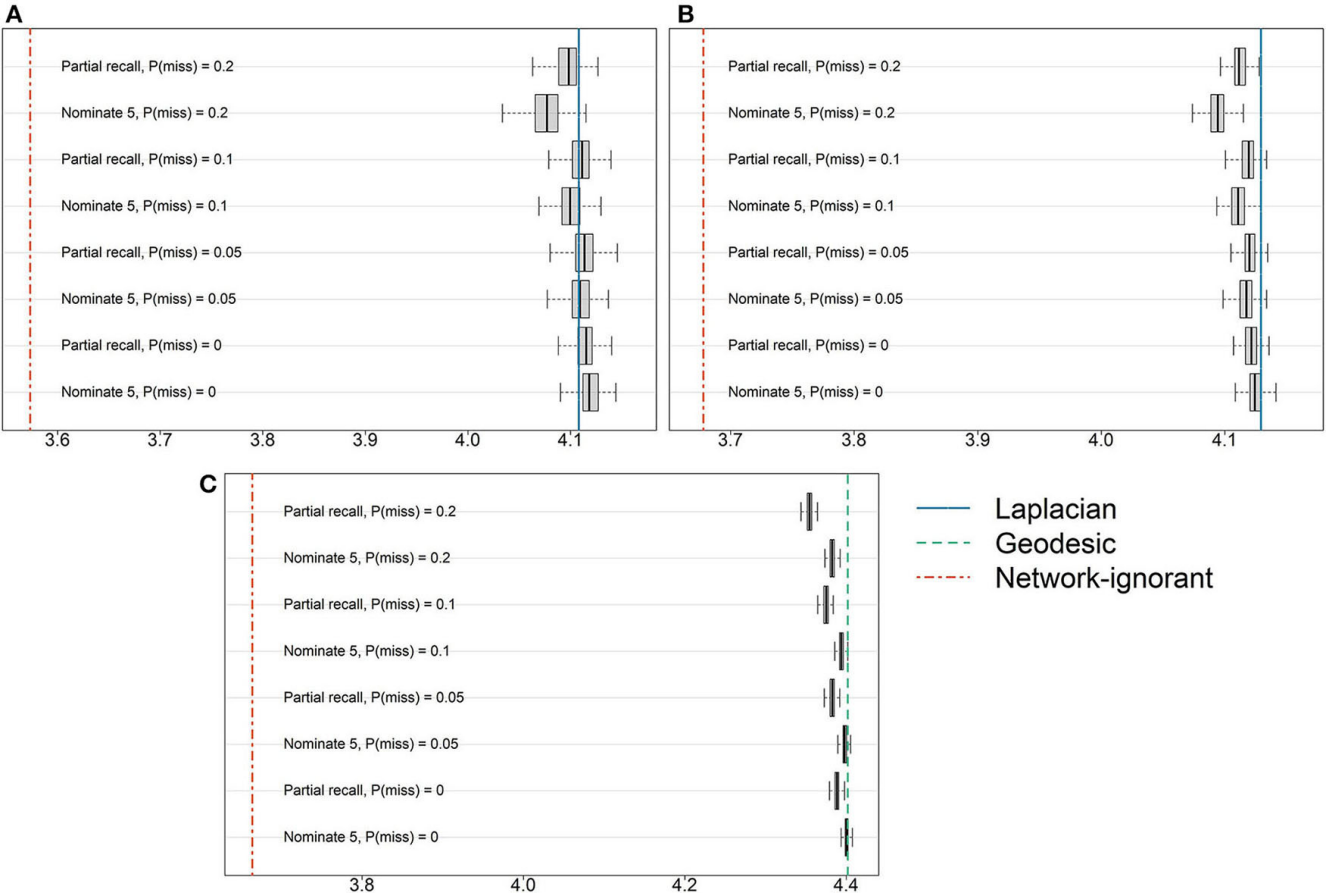
Results from introducing missingness into the **(A)** AH495, **(B)** AH2587, or **(C)** ERGM10000 network by simulating two common network survey tools and varying the level of non-response. The horizontal axis corresponds to *Q*(*Z*), and vertical lines show either the average of 50 random pool assignments or results based on the true underlying network.

Figure 2 provides the results from perturbing the network by introducing missingness due to survey design and non-response rates. For reference, the oracle results using either Algorithm 1 or Algorithm 2 are presented as a vertical line, as are the results from random pool assignments. All results correspond to the optimal *K* given above. There is no clear pattern of superiority when comparing the two survey designs, nominate-5 and partial recall. While the results deteriorate somewhat as the non-response rate increases, these decreases are very marginal compared to random pool assignments that do not leverage the network information.

When our algorithms were run on a personal computer with an Intel(R) Core(TM) i7-9850H CPU 2.60GHz processor, we obtained the computation times provided in Table 1. These results indicate that our approach can feasibly be applied to even large organizations.

**Table 1:** Computational time in seconds to run Algorithms 1, 2.

**Network**	**Laplacian**	**Geodesic**
AH495	2.95	0.04
AH2587	1080.22	1.07
ERGM10000	NA	16.42

## 4. Discussion

Regular universal screening can play an important role in infection control. The cost of implementing this strategy, however, can be out of reach for many organizations. Pooling tests and only testing individuals should their pool test positive leads to fewer overall tests being conducted, thereby lowering the resource burden to a more manageable level.

While the extant literature on pooled testing is vast, algorithms that aim at finding the optimal pool size ignore the fact that in the context of infectious disease there is an underlying transmission network that makes the individuals to be pooled not independent. We have shown that by utilizing the underlying network, the cost savings provided by pooled testing can be further increased.

In real applications, the true underlying contact network that leads to transmission events is of course unknown. We have shown, however, that using easily implemented survey tools to collect contact information can provide enough information about the network to yield results nearly equivalent to when the true network is known. Furthermore, our methods are robust to high non-response rates.

## Data Availability Statement

Publicly available datasets were analyzed in this study. These data can be found here: https://github.com/Z-co/networkdata.

## Author Contributions

The author confirms being the sole contributor of this work and has approved it for publication.

## Funding

This study was supported by the US Centers for Disease Control and Prevention (5 U01 CK000531-02) as part of the MInD-Healthcare Program.

## Conflict of Interest

The author declares that the research was conducted in the absence of any commercial or financial relationships that could be construed as a potential conflict of interest.

## Publisher's Note

All claims expressed in this article are solely those of the authors and do not necessarily represent those of their affiliated organizations, or those of the publisher, the editors and the reviewers. Any product that may be evaluated in this article, or claim that may be made by its manufacturer, is not guaranteed or endorsed by the publisher.
